# Circulating Chromogranin A Is Cleaved Into Vasoregulatory Fragments in Patients With Pancreatic Ductal Adenocarcinoma

**DOI:** 10.3389/fonc.2020.613582

**Published:** 2020-12-23

**Authors:** Michele Reni, Valentina Andreasi, Anna Maria Gasparri, Erica Dugnani, Barbara Colombo, Marina Macchini, Mimma Bianco, Alice Dallatomasina, Antonio Citro, Emma Assi, Maria Pia Protti, Antonio Esposito, Massimo Falconi, Flavio Curnis, Lorenzo Piemonti, Angelo Corti

**Affiliations:** ^1^ Department of Medical Oncology, IRCCS San Raffaele Scientific Institute, Milan, Italy; ^2^ Division of Experimental Oncology, IRCCS San Raffaele Scientific Institute, Milan, Italy; ^3^ Pancreatic Surgery Unit, IRCCS San Raffaele Scientific Institute, Milan, Italy; ^4^ Faculty of Medicine and Surgery, Vita-Salute San Raffaele University, Milan, Italy; ^5^ Diabetes Research Institute, IRCCS San Raffaele Scientific Institute, Milan, Italy; ^6^ Division of Immunology, IRCCS San Raffaele Scientific Institute, Milan, Italy; ^7^ Experimental Imaging Center, IRCCS San Raffaele Scientific Institute, Milan, Italy

**Keywords:** pancreatic ductal adenocarcinoma, chromogranin A, proteolytic processing, plasminogen activators, vasostatin-1

## Abstract

Chromogranin A (CgA), a secretory protein released in the blood by the neuroendocrine system, consists of a mixture of full-length molecules and fragments endowed of vasoregulatory activity. The extent and the role of CgA fragmentation were investigated in patients with locally advanced or metastatic pancreatic ductal adenocarcinoma (PDAC, n=172). Multivariate analysis showed that full-length CgA was associated with better progression free and overall survival, whereas CgA C-terminal fragmentation was associated with worse prognosis. In vitro studies showed that PDAC cells can promote the cleavage of CgA C-terminal region by activating plasminogen to plasmin. Limited digestion of full-length CgA with plasmin abolished its anti-angiogenic activity and generated pro-angiogenic molecules. The fragmentation of CgA C-terminal region was increased also in murine models of PDAC. In these models, the inhibition of CgA fragmentation with aprotinin, an inhibitor of plasmin and other serine proteases, or the blockade of pro-angiogenic fragments with specific antibodies inhibited the growth of PDAC implanted subcutaneously in mice. Finally, administration of full-length CgA to mice bearing orthotopic PDAC reduced tumor perfusion, as measured by contrast-enhanced ultrasound. These findings suggest that PDAC can promote the cleavage of circulating CgA C-terminal region to generate fragments that regulate the tumor vascular biology and that may represent new potential therapeutic targets.

## Introduction

Pancreatic ductal adenocarcinoma (PDAC), the most common type of pancreatic cancer, is an aggressive malignancy arising from the exocrine epithelial component of the pancreas (1). PDAC currently represents the fourth cause of cancer-related death (2). Despite the increasing incidence, the prognosis of this neoplasm is still poor and the therapeutic options are very limited (3, 4). Thus, studies aimed at investigating the mechanisms of PDAC growth and the identification of new prognostic markers and therapeutic targets are of great experimental and clinical relevance.

A growing body of evidence suggests that circulating chromogranin A (CgA), an acidic secretory protein, may represent an important regulating factor of the vascular biology of solid tumors (5–8). This protein is stored in the secretory granules of many normal neuroendocrine cells and neurons (9, 10) and is exocytotically released in circulation, reaching 0.2-1 nM in healthy subjects (7, 11). Higher CgA levels have been detected in patients with neoplastic, cardiovascular, renal, gastrointestinal, and inflammatory diseases, with, in some cases, important diagnostic and prognostic implications (9, 11). Structural studies have shown that circulating CgA consists of a mixture of full-length CgA (CgA_1-439_) and CgA-derived fragments of different size, including peptides lacking part or the entire CgA_410-439_ region and shorter fragments (12). CgA_1-439_ and some of its fragments (e.g., vasostatin-1, catestatin and serpinin) can exert a variety of biological functions in the regulation of cardiovascular system, metabolism and innate immunity (13). Regarding their vascular functions, recent studies have shown that CgA_1-439_ can exert anti-angiogenic effects at physiological concentrations (5, 6, 8). An active anti-angiogenic site is located in its C-terminal region, whereas a latent (or less active) site is located in the N-terminal region, this site requiring cleavage of the Q_76_-K_77_ peptide bond to generate CgA_1-76_ (vasostatin-1), an anti-angiogenic fragment (5). In contrast, cleavage of R_373_-R_374_ site in the C-terminal region generates CgA_1-373_, a pro-angiogenic fragment (5, 14). The pro-angiogenic activity of CgA_1-373_ requires the binding of its Pro-Gly-Pro-Leu-Arg_373_ site (PGPQLR_373_) to neuropilin-1: accordingly, antibodies against this site (which is cryptic in CgA_1-439_) can reduce angiogenesis and tumor growth in various animal models of solid tumors (8). Further processing of CgA_1-373_ to CgA_1-372_ and consequent loss of the C-terminal arginine, which can occur in the blood, causes loss of neuropilin-1 binding and gain of anti-angiogenic activity (8). Thus, cleavage of circulating CgA in tumors and then in the blood may represent a complex mechanism for the spatio-temporal regulation of the tumor vascular biology. According to this view, a significant correlation between CgA fragmentation at the R_373_-R_374_ site and tumor microvessel density has been observed in the bone marrow of patients with multiple myeloma (12). However, the prognostic value of CgA cleavage on tumor progression and patient survival still remains unexplored. These notions prompted us to investigate the extent of CgA N- and C-terminal proteolytic processing in patients diagnosed with PDAC and to explore its prognostic value and function. We show that cleavage of circulating CgA C-terminal region is increased in patients and that CgA cleavage predicts worse progression-free and overall survival. Furthermore, using *in vitro* and *in vivo* models of PDAC, we provide data suggesting a role of the plasminogen activator-plasmin system in CgA cleavage and a function of CgA fragmentation in the regulation of PDAC vascular biology.

## Materials and Methods

### Patients and Plasma Samples

Patients with cyto/histologically proven stage III or IV untreated PDAC were enrolled in this study. Additional eligibility criteria included: age 18–70 years and Karnofsky performance status >60, or 71–75 years and Karnofsky performance status >70; absolute neutrophil count ≥1500/mm^3^, platelet count ≥100,000/mm^3^, hemoglobin ≥10 g/dl; serum creatinine ≤1.5 mg/dl; total bilirubin ≤1.5 mg/dl; transaminases ≤3 times the upper limit of normal. This study was approved by the local ethics committee (OSR-Prot. PACT-19) and was conducted at the San Raffaele Hospital of Milan (authorized organization) following the principles of good clinical trial practice according to the Declaration of Helsinki. A written informed consent was obtained from each patient.

Five milliliters of peripheral blood were collected at baseline, before the beginning of chemotherapy, in a sterile test-tube and centrifuged at 2,500xg for 10 min. Plasma samples were stocked in 0.5 ml aliquots in cryovials and stored at -80°C until measurement.

### Recombinant CgA, Fragments, Antibodies, and Immunoassays

Human CgA_1-439_ and CgA_1-373,_ were prepared by recombinant DNA technology and characterized by mass spectrometry analysis (sequence), SDS-PAGE, and western blotting analyses as previously reported (5, 6, 12, 15). Polyclonal and monoclonal antibodies against various epitopes of CgA located in the N-terminal, central and C-terminal regions of CgA have been prepared as described in **Supplementary Materials**. Rabbit IgGs against the CgA_368-373_ epitope (PGPQLR) were purified by affinity chromatography on protein A-sepharose as previously described (8). CgA_1-439_ and fragments were detected in plasma samples using five sandwich ELISAs based on antibodies against different CgA regions (see **Supplementary Methods** and **Figure S1** for schematic representation of antibody epitopes and assays, and **Table S1** for analyte specificity of each assay) (8, 12). These assays can selectively detect: a) full-length CgA (CgA_1-439_−ELISA); b) full-length CgA with or without the C-terminal sequence 437-439 (CgA_1-436/439_−ELISA); c) CgA_1-372_ and CgA_1-373_ (CgA_1-372/373_−ELISA); d) CgA_1-76_ (CgA_1-76_−ELISA); e) full-length CgA and fragments containing the N-terminal region plus part or the entire central and C-terminal regions, but not CgA_1-76_ (defined here as “CgA_total_”) (CgA_total_−ELISA) (12).

The cumulative amounts of large fragments lacking the region 410-430 region (e.g., CgA_1-409_, CgA_1-394_, CgA_1-373_, plus other potential fragments with unknown C-terminus), were calculated as the difference between CgA_total_ and CgA_1-436/439_ and collectively called CgA_1-x_.

### Cells

Human PDAC cells BxPC-3, Hs766T, MiaPaCa-2, PT45, A8184, murine PDAC cells DT6606, cancer-associated fibroblasts, and human monocytes were obtained and cultured as described in **Supplementary Methods**.

### Endothelial Spheroid Capillary Sprouting Assay

Endothelial cell spheroid assays were performed using human umbilical vein endothelial cells as described (6, 8) (see also **Supplementary Methods**).

### *In Vivo* Studies in PDAC Animal Models

All procedures on mice were approved by the San Raffaele Institutional Animal Care and Use Committee, and by the Ministero della Salute of Italy (Author. 596/2016PR and 637). The study was performed at the San Raffaele Hospital (authorized organization) according to institutional guidelines and in compliance with national and international law and policies. Eight-week-old Rag2-/-γc-/- male mice (CIEA and Taconic, Japan) were challenged subcutaneously with 5x10^6^ BxPC-3 or HS766T cells, resuspended in 0.9% sodium chloride and mixed with Cultrex^®^ Basement Membrane Matrix. Blood samples from animals were collected weekly until day 35 and, in the case of HS766T-tumor bearing mice, at day 68. Eight-week-old C57BL/6/N female mice (Charles River Laboratories Italia S.p.A., Calco, Italy) were challenged with subcutaneous injection of 2x10^6^ Panc02 cells in 0.9% sodium chloride. Tumor growth was monitored weekly by measuring tumor volumes with calipers. The animals were sacrificed when tumor diameter reached 1–1.5 cm.

Syngeneic eight-week-old C57BL/6N male mice (Charles River) were orthotopically injected with DT6606 PDAC cells. To this aim 1x10^6^ cells/50 µl in phosphate-buffered saline containing 25% Growth Factor Reduced Matrigel (BD Biosciences) were injected into the pancreas of mice as previously described, with minor modification (16). Mice were treated with CgA (1.5 µg/mouse, i.p. twice a week, in 0.9% sodium chloride) or with vehicle alone. Tumor perfusion was monitored by contrast-enhanced ultrasound analysis using a high-performance ultrasonographic scanner designed for small animal imaging (Vevo 2100; Visual Sonics) as described (6, 17).

### Statistical Methods

Study endpoints were progression-free and overall survival. Cox proportional hazards models with backward selection were used to estimate independent predictors of progression-free and overall survival. Continuous variables were compared using Mann-Whitney U test or Student’s *t*-test, as appropriate. Statistical analysis was performed using the SPSS 25.0 for Mac software (SPSS Inc., Illinois, USA). *P* values <0.05 were considered significant.

## Results

### Full-Length CgA and Various Fragments Are Present in the Peripheral Blood of Healthy Subjects and Patients with PDAC

Plasma levels of CgA and its fragments were measured in 172 patients with locally advanced or metastatic PDAC (**Table S2**) and 37 age-matched healthy subjects. To this aim we used five different ELISAs (CgA_1-439_–, CgA_1-436/439_–, CgA_1-372/373_–, CgA_1-76_–, and CgA_total_–ELISA) based on antibodies against different CgA epitopes. These assays and the analytes detected are described in **Table S1** and schematically represented in **Figure S1**.

The results **(** **Table 1** **)** suggest that in heathy subjects circulating CgA consists of a mixture of full-length CgA_1-439_ (median 0.19 nM) and fragments (CgA_total_, 0.77 nM). CgA_1-436/439_, which include large molecules containing N-terminal, central and part or entire C-terminal region, accounted to about a half of CgA_total_, suggesting that a conspicuous amount of the circulating CgA (about a half) consisted of CgA_1-x_, a family of large fragments lacking the entire C-terminal region, some with an undefined C-terminus. Of note, the CgA_1-372/373_ component of the CgA_1-x_ family was undetectable in normal subjects **(** **Table 1**), suggesting that most CgA_1-x_ fragments in healthy subjects were different from CgA_1-372/373_. The results also show that the N-terminal fragment CgA_1-76_ was present in the blood of healthy subjects, suggesting that partial cleavage of CgA N- and C-terminal regions occurs in healthy conditions.

**Table 1:** Plasma levels of CgA and fragments in patients with pancreatic ductal adenocarcinoma (PDAC) and in healthy subjects.

	Healthy subjects *n* = 37	All patients *n* = 172	*P value*
**CgA_total_ (nM)** Median 25^th^–75^th^ Min-max	0.77 0.62–0.99 0.39–2.17	0.92 0.61–1.58 0.22–14.28	*0.101*
**CgA_1-439_ (nM)** Median 25^th^–75^th^ Min-max	0.19 0.11–0.29 0.03–0.64	0.13 0.10–0.17 0.04–0.99	***0.013***
**CgA_1-436/439_ (nM)** Median 25^th^–75^th^ Min-max	0.35 0.30–0.49 0.09–0.78	0.47 0.32–0.78 0.05–3.71	***0.001***
**CgA_1-x_ (nM)** Median 25^th^–75^th^ Min-max	0.41 0.32–0.62 0.15–1.39	0.44 0.26–0.87 0.001–11.93	*0.775*
**CgA_1-372/373_ (nM)** Median 25^th^–75^th^ Min-max	0.05 0.05–0.06 0.03–0.07	0.16 0.11–0.26 0.04–6.73	***<0.0001***
**CgA_1-76_ (nM)** Median 25^th^–75^th^ Min-max	0.33 0.28–0.39 0.11–0.48	0.32 0.21–0.45 0.01–3.61	*0.916*
**CgA_1-x_/CgA_total_** Median 25^th^–75^th^ Min-max	0.55 0.50–0.61 0.26–0.84	0.52 0.42–0.61 0.001–0.97	***0.047***
**CgA_1-439_/CgA_total_** Median 25^th^–75^th^ Min-max	0.24 0.15–0.39 0.04–0.50	0.12 0.09–0.20 0.01–0.49	***<0.0001***

Bold text indicate statistically significant values.

**Table 1** also shows the results of assays performed on plasma samples from PDAC patients. Although CgA_total_ was not significantly different in healthy subjects and patients, some differences were observed in terms of fragmentation, particularly at the C-terminal region. Indeed, full-length CgA_1-439_ was significantly decreased in patients, while CgA_1-372/373_ was significantly increased (**Table 1** and **Figure S2**). In contrast, similar levels of CgA_1-76_ were observed in patients and controls. These data suggest that increased CgA fragmentation at its C-terminal region, but not at its N-terminal regions, occurred in patients. Of note, a modest non-significant increase of CgA_1-x_ was observed, suggesting that CgA_1-372/373_ (which belongs to the CgA_1-x_ family) mainly derive from another larger CgA_1-x_ fragment and in part from full-length CgA. These results, overall, indicate that an important change in the balance between full-length CgA and fragments, particularly those lacking the C-terminal region, occurred in PDAC patients.

### Proton Pump Inhibitor Administration Is Not the Cause of Increased CgA Fragmentation

Considering that 56% of the PDAC patients were treated with proton pump inhibitors, a class of drugs that may induce the release of CgA from enterochromaffin-like cells of the gastric mucosa (18, 19), the impact of this treatment on circulating CgA was then assessed. Of note, CgA_1-436/439_ levels were significantly higher in patients treated with proton pump inhibitors, compared to untreated patients (0.49 *vs* 0.42 nM, *P*=0.042), while CgA_1-372/373_ were similar (**Table S3**). Furthermore, the CgA_1-x_/CgA_total_ ratio, which represents a sort of C-terminal fragmentation index, was significantly lower in proton pump inhibitors-treated patients (0.49 *vs* 0.54, *P*=0.032). Thus, proton pump inhibitor administration was not the cause of increased CgA fragmentation observed in these patients.

### Enhanced C-Terminal Cleavage of CgA Predicts Progression-Free and Overall Survival in PDAC Patients

After blood collection, 148 patients (86%) received a combination of cisplatin, epirubicin, capecitabine and gemcitabine, and 24 patients (14%) received the same association with docetaxel in place of epirubicin, for 6 months or until disease progression. Follow up data, progression-free and overall survival are shown in **Table S4**. Patients with locally advanced PDAC had a median progression-free and overall survival of 10 and 18 months, whereas patients with metastatic disease had a median progression-free and overall survival of 6 and 10 months, respectively. A univariate Cox-regression analysis showed that stage of disease, Karnofsky performance status, and baseline CA19.9, but not CgA and its fragments, were significantly associated with progression-free and overall survival (**Table S5**). However, multivariate analysis showed that CgA and its fragments, in addition to age, stage, Karnofsky Performance Status, baseline CA19.9, and chemotherapy regimen, were all significantly associated with progression-free and overall survival (**Table 2**). Full-length CgA (CgA_1-439_, min-max 0.04–0.99 nM) was a strong predictor of better outcome, both in terms of progression-free survival (hazard ratio: 0.040, *P*<0.0001) and overall survival (hazard ratio: 0.025, *P*<0.0001). These data indicate that every unit increase of this analyte provides a 96% risk reduction for progression and a 97.5% risk reduction for death due to any cause. In contrast, a high CgA_1-x_/CgA_total_ ratio, an index of CgA fragmentation in the C-terminal region, was a strong predictor of poor overall survival (hazard ratio: 3.533, *P*=0.015), with every unit of this index providing a 253% risk increase for death due to any cause. An association with a worse prognosis was found also for CgA_1-372/373_ (progression-free survival, hazard ratio: 1.522, *P*=0.020) and for CgA_1-436/439_ (overall survival, hazard ratio: 1.595, *P*=0.006). No association was observed with CgA_1-76_ or CgA_1-76_/CgA_total_ ratio. In conclusion, the results of the immunoassays suggest that enhanced fragmentation of circulating CgA and consequent decrease of full-length CgA and increase of fragments lacking the C-terminal region is associated with worse outcome in PDAC patients.

**Table 2:** Multivariate Cox-regression analysis evaluating predictors of progression-free survival and overall survival (OS) in patients with locally advanced or metastatic pancreatic ductal adenocarcinoma (PDAC) (n=172).

Variable	Progression-free survival			Overall survival		
	HR^a^	95% C.I.	*P*	HR^a^	95% C.I.	*P*
**Sex** Female Male	–	–	–	1 1.312	- 0.956-1.801	*0.093*
**Age** ≤ 60 years > 60 years	1 0.602	- 0.437–0.830	***0.002***	1 0.685	- 0.497–0.943	***0.020***
**Stage** III IV	1 2.821	- 1.994–3.992	***<0.0001***	1 2.140	- 1.534–2.985	***<0.0001***
**Karnofsky performance status**	0.960	0.940–0.980	***<0.0001***	0.965	0.945–0.985	***0.001***
**CA 19.9**	1.017	1.005–1.030	***0.007***	1.022	1.009–1.034	***<0.0001***
**Chemotherapy regimen** PDXG PEXG	1 1.978	- 1.219–3.209	***0.006***	1 1.550	- 0.952–2.523	*0.078*
**Proton pump inhibitors** No Yes	–	–	*–*	–	–	–
**CgA_total_**	–	–	*–*	–	–	–
**CgA_1-439_**	0.040	0.007–0.239	***<0.0001***	0.025	0.003–0.174	***<0.0001***
**CgA_1-436/439_**	1.400	0.960–2.041	*0.080*	1.595	1.143–2.225	***0.006***
**CgA_1-x_**	–	–	*–*	–	–	–
**CgA_1-372/373_**	1.522	1.069–2.167	***0.020***	–	–	–
**CgA_1-76_**	–	–	***–***	–	–	–
**CgA_1-x_/CgA_total_**	2.384	0.891–6.380	*0.084*	3.533	1.276–9.782	***0.015***
**CgA_1-439_/CgA_total_**	–	–	–	–	–	–

HR, hazard ratio; C.I., confidence interval; PDXG, cisplatin+docetaxel+capecitabine+gemcitabine; PEXG, cisplatin+epirubicin+capecitabine+gemcitabine.

^a)^HR for every increase of: 10% (performance status); 1,000 U/ml (CA 19.9); 1 nM (CgA_total_, CgA_1-439_, CgA_1-436/439_, CgA_1-x_, CgA_1-372/373_, CgA_1-76_); 1 unit (CgA_1-x_/CgA_total_, CgA_1-439_/CgA_total_).

Bold text indicate statistically significant values.

### Pancreatic Cancer Cell Lines Promote CgA C-Terminal Cleavage By Activating Plasminogen to Plasmin

**Figure 1:**
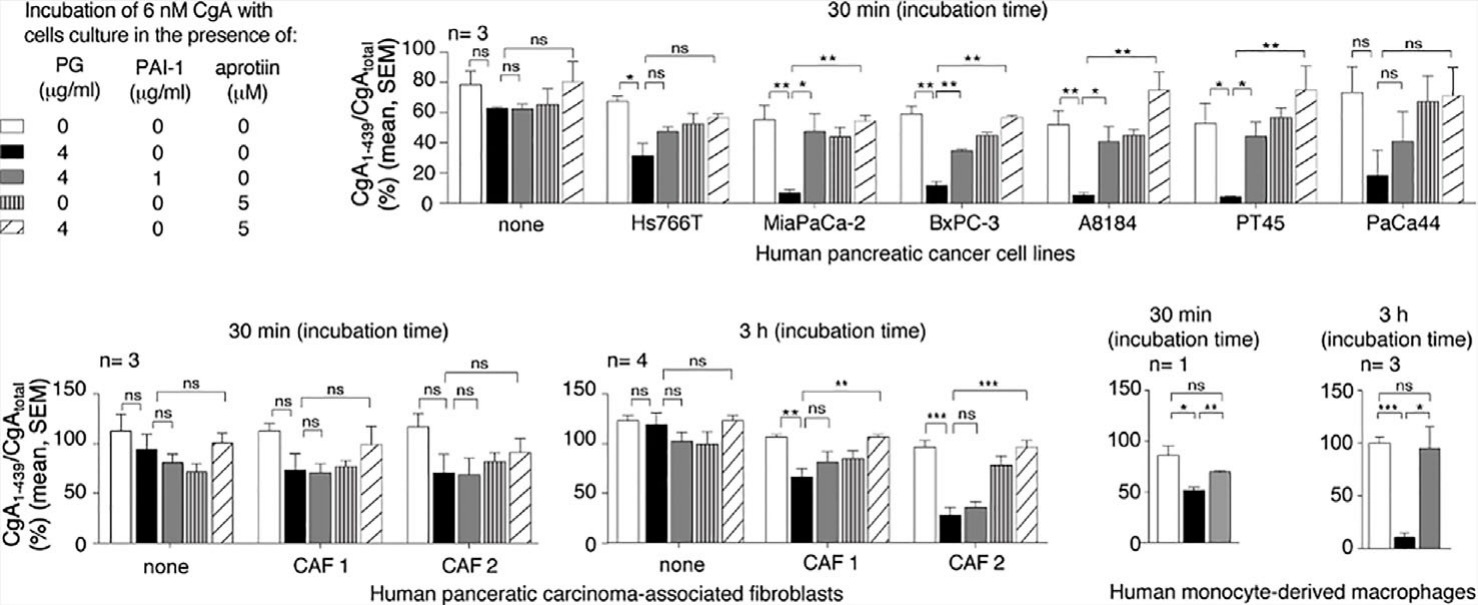
Effect of pancreatic ductal adenocarcinoma (PDAC) cells, pancreatic adenocarcinoma-associated fibroblasts and human monocyte-derived macrophages on CgA cleavage. Human PDAC cells (Hs766T, MiaPaCa-2, BxPC-3, A8184, PT45, PaCa44), PDAC-associated fibroblasts (CAF1 and CAF2) and monocyte-derived macrophages (5x10^5^ cells/well of 96-well plates) were cultured for 24–48 h at 37°C. After washing, recombinant CgA_1-439_ (6 nM), plasminogen (PG) (4 µg/ml), plasminogen activator inhibitor-1 (PAI-1) (1 µg/ml) and aprotinin (5 µM) were added to cell culture medium containing 0.5% of bovine serum albumin, and further incubated for 30 min or 3 h at 37°C, as indicated. The supernatants were collected and analysed by CgA_1-439_– and CgA_total_–ELISAs. The CgA_1-439_/CgA_total_ ratio, which represent an index of CgA integrity, is shown. Bars (mean ± SEM); *P < 0.05; **P < 0.01; ***P < 0.001 (*t*-test, two tails); *ns*, not significant.

The mechanism of CgA fragmentation was then investigated. To investigate the role of cancer cells on CgA fragmentation we incubated recombinant CgA_1-439_ in the presence or absence of various human PDAC cell lines (Hs766T, MiaPaCa-2, BxPC-3, A8184, PT45, and PaCa44 cells) or murine Panc02 cells. CgA fragmentation in cell supernatants was monitored using CgA_1-439_– and CgA_total_–ELISA. After 30 min of incubation, little or no changes in CgA_1-439_/CgA_total_ ratio were observed with all cell lines tested, arguing against a direct role of PDAC cells in CgA fragmentation. However, when plasminogen was added to the cultures, a marked reduction of CgA_1-439_/CgA_total_ ratio was observed with the majority of PDAC cell lines (**Figure 1**). The addition of plasminogen activator inhibitor 1 (PAI-1) or of aprotinin (an inhibitor of plasmin and other serine proteases) significantly inhibited the degradation of CgA by most PDAC cell lines, pointing to a crucial role of plasminogen activators and plasmin in this process (**Figure 1**). These data suggest that PDAC cells can promote CgA cleavage by releasing plasminogen activators and, consequently, by converting plasminogen into active plasmin. Similar experiments were performed with human cancer-associated fibroblasts isolated from two PDAC (CAF-1 and CAF-2) or with human monocyte-derived macrophages. No significant degradation was observed with cancer-associated fibroblasts after 30 min, even in the presence of plasminogen (**Figure 1**). However, partial degradation was observed after 3 h of incubation, suggesting that also these cells may contribute to CgA degradation, although less efficiently than cancer cells. The same applies also for human monocyte-derived macrophages (**Figure 1**). Considering that both cell types may represent a large component of the tumor tissues, their contribution to CgA degradation could also be relevant.

### The C-Terminal Region of CgA Is More Prone to Plasmin Cleavage Than the N-Terminal Region

**Figure 2:**
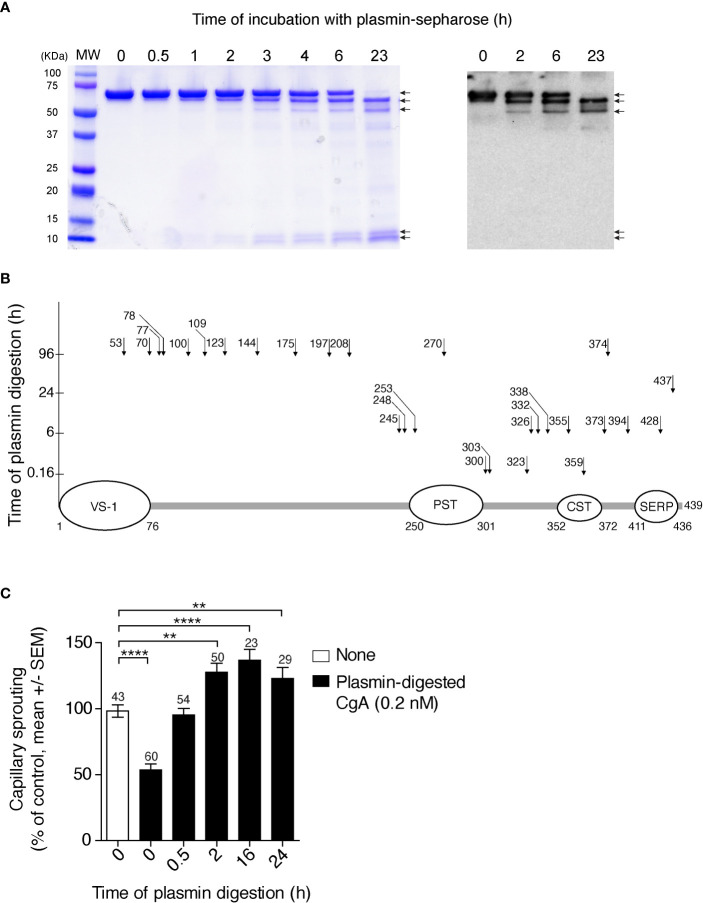
Time course of CgA cleavage by plasmin, cleavage sites, and anti-/pro-angiogenic activity before and after cleavage. **(A)** SDS-PAGE and western blotting analysis of CgA before and after digestion with plasmin. Recombinant full-length CgA_1-439_ (9 µM, 100 µl) was incubated with plasmin-Sepharose beads (BioVision) (10 µl) in 0.05 M sodium chloride, 0.15 M sodium phosphate buffer, pH 7.3, for the indicated time. At various time points, the beads were removed, by centrifugation, and the supernatants were analysed by SDS-PAGE (Coomassie dye staining) (*left panel*) and western blotting with the αFRs antiserum against the central region of CgA (*right panel*). MW, molecular markers. **(B)** Schematic representation of full-length CgA and its plasmin cleavage sites (*arrows* and number of cleaved residue) identified by mass spectrometry analysis of the low molecular weight fragments obtained by limited and extensive digestion of CgA with plasmin-sepharose. The time of digestion is indicated on the y axis. As shown the C-terminal region is more prone to proteolysis than the N-terminal region, the latter requiring 96 h of incubation for cleavage. The regions corresponding to vasostatin-1 (VS-1); pancreastatin (PST); catestatin (CST); and serpinin (SERP) are indicated. **(C)** Effect of plasmin cleavage on the anti-angiogenic activity of full-length CgA, as measured using the endothelial-spheroid capillary-sprouting assay. In a separate digestion experiment, plasmin-Sepharose was removed by centrifugation at the indicated time points; the supernatants, diluted to 0.2 nM CgA, were incubated overnight with endothelial cell spheroids. Each bar represents the number of capillaries sprouting from the spheroids (mean ± SEM, see Methods). The number of spheroids analysed for each time point is indicated on each bar. **P <0.01; ****P <0.0001 (t-test, two tails).

To gain more information on the sites cleaved by plasmin, full-length CgA was incubated with a small amount of this enzyme for various time intervals. The products were then analysed by SDS-PAGE and mass spectrometry. After 6 h of incubation, SDS-PAGE showed major bands corresponding to undigested CgA (70 kDa), large fragments of about 60–65 kDa, and ~10 kDa small fragments (**Figure 2A**, left). The large 60–65 kDa fragments, but not the ~10 kDa fragments, were recognized by an antiserum against the central region of CgA (αFRs), by western blot analysis (**Figure 2A**, right), pointing to N- or C-terminal cleavage. The large fragments were not detectable by mass spectrometry for unknown reasons. However, this assay could identify a series of other sub-fragments, present in smaller amounts, which were consistent with limited digestion at various sites in the C-terminal region (residues 246–439), but not in the N-terminal region (1–245) of CgA (**Figure 2B**). Notably, fragmentation of the N-terminal part required longer time to occur (96 h), which implies that the N-terminal part is more resistant than the C-terminal part to plasmin digestion. Taking into consideration the molecular weight of the major fragments (60–65 kDa, determined by SDS-PAGE) and the cleavage sites identified by mass spectrometry after 6 h, we estimate that the large fragments observed by SDS-PAGE and western blotting correspond to CgA cleavage in the C-terminal region, presumably at residues 394, 373, and/or 355/359. Indeed, cleavage at these sites is expected to shorten the molecule of 45–84 residues (~5-10 kDa), as observed by SDS-PAGE. In conclusion, these results suggest that limited digestion with plasmin can generate large fragments ending with residues 394, 373, and/or 355/359, which are all components of the CgA_1-x_ family. This view is in keeping with previous observation showing that an antibody specific for the PGPQLR epitope of CgA_1-373_ (cryptic in intact CgA) could recognize a 60–62 kDa band by western blotting analysis of CgA digested with plasmin for 2–6 h (12), likely corresponding to the CgA_1-373_ analyte detected in plasma samples of PDAC patients (**Figure S2**).

### Limited Digestion With Plasmin Converts Anti-Angiogenic Full-Length CgA Into Pro-Angiogenic Fragments

We have previously shown that CgA_1-439_ and CgA_1-373_ are anti- and pro-angiogenic molecules, respectively (5). To assess the impact of plasmin digestion on the biological activity of CgA_1-439,_ we analysed its anti-/pro-angiogenic activity before and after exposure to plasmin, using the endothelial cell spheroid capillary sprouting assay. As expected, limited digestion of CgA_1-439_ caused loss of anti-angiogenic activity and gain of pro-angiogenic effects, as indicated by the lower number of capillary-like sprouts induced by CgA before digestion and by the higher number of sprouts after 2–16 h of incubation, compared to untreated spheroids (**Figure 2C**). Extensive digestion for 24 h reduced the pro-angiogenic effects. These results suggest that limited digestion of CgA C-terminal region by plasmin is associated with a marked change of function, from anti- to pro-angiogenic.

### CgA C-Terminal Fragmentation Is Increased in Mouse Models of PDAC

**Figure 3:**
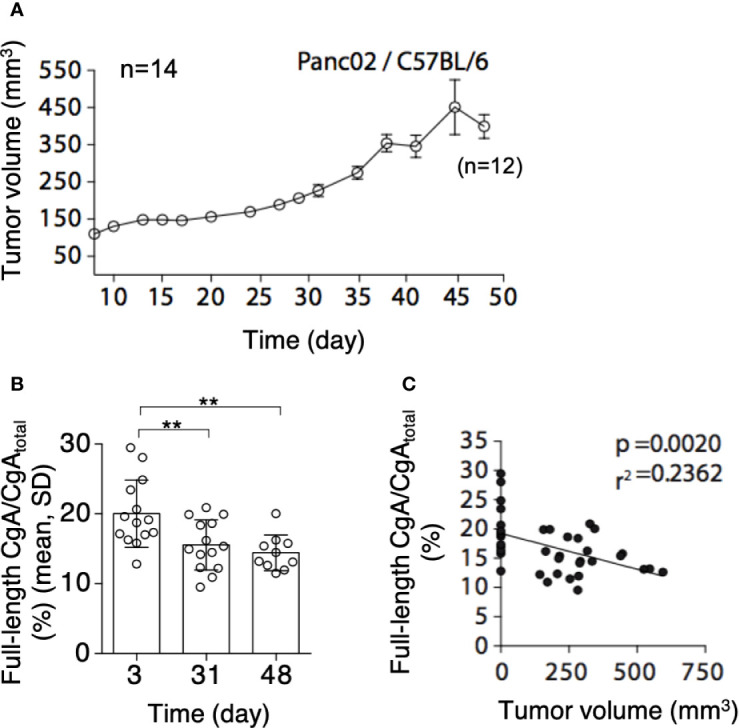
Tumor growth and CgA fragmentation in the murine Panc02 model. **(A)** Growth rate of Panc02 tumors implanted subcutaneously in syngeneic mice. Tumor volumes were measured with calipers (mean± SEM, n=14). As 2 mice died at day 48, the data reported at this time point represent the tumor volumes of 12 mice. **(B)** Endogenous full-length CgA/CgA_total_ ratio (an index of CgA integrity) as measured at day 3, 31 and 48 after tumor implantation. Endogenous full-length CgA and CgA_total_ were measured by ELISA of plasma samples collected at day 3 and 31 (n=14). Only 10 plasma samples (from 12 surviving mice) were collected in sufficient amount for analysis at day 48. Bars (mean ± SD); **, *P <*0.01 (unpaired *t*-test). **(C)** Correlation between full-length CgA/CgA_total_ ratio and tumor volumes at day 3, 31, and 48. The tumor volumes at day 3, undetectable, were considered <1 mm^3^.

**Figure 4:**
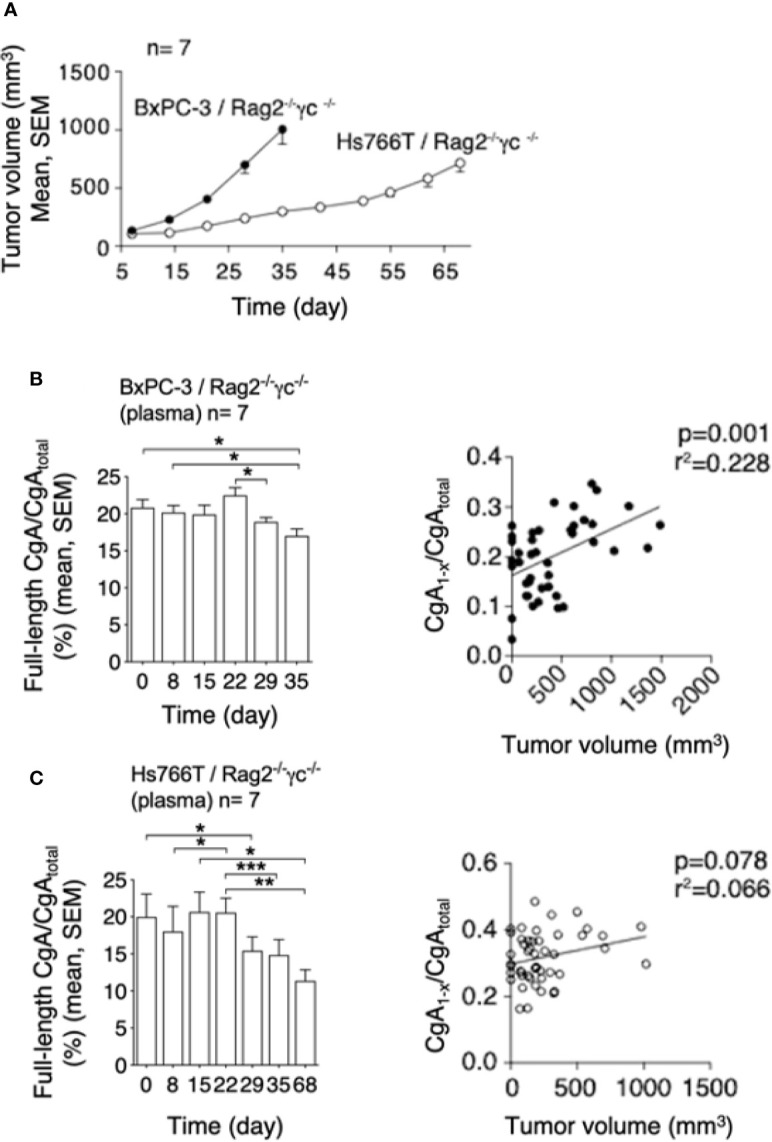
Tumor growth and CgA fragmentation in the human BxPC-3 and Hs766T models. **(A)** Growth rate of BxPC-3 and Hs766T tumors implanted subcutaneously in Rag2^-/-^γc^-/-^ mice (n=7). Tumor growth was monitored with calipers. **(B, C)** *Left panels*: endogenous full-length CgA/CgA_total_ ratio (an index of CgA integrity) at the indicated time points after tumor implantation. Circulating full-length CgA and CgA_total_ were measured by ELISA of plasma samples collected at the indicated time. Bars (mean ± SEM); *P <0.05; **P <0.01; ***P <0.001 (paired *t*-test). *Right panels*: correlation between CgA_1-x_/CgA_total_ ratio (an index of CgA C-terminal degradation) and tumor volume, as measured in various mice at various times.

To assess whether PDAC cells can cause CgA fragmentation also *in vivo*, we monitored CgA levels in plasma samples obtained from C57BL/6 mice bearing subcutaneous Panc02 tumors (**Figure 3A**), using CgA_1-439_– and CgA_total_–ELISA (taking advantage from antibody cross-reactivity with the murine full-length CgA_1-445_ and CgA_total_). A significant decrease of full-length CgA/CgA_total_ ratio was observed from day 3 to day 31-48 after tumor implantation (**Figure 3B**, left). This finding is consistent with an increased proteolytic processing of circulating CgA upon tumor growth. Of note, the full-length CgA/CgA_total_ ratio negatively correlated with tumor volume (**Figure 3B**, right), likely because large amounts of protease are produced by large tumors. Similar results were observed also in other models based on human BxPC-3 or Hs766T PDAC cells implanted subcutaneously in Rag2^-/-^γc^-/-^ mice (**Figures 4A–C**). Interestingly, in the BxPC-3 model the tumor burden positively correlated with CgA_1-x_/CgA_total_ ratio (**Figure 4B**, right), pointing again to enhanced C-terminal fragmentation by larger tumors. These findings suggest that also in these models tumor growth was associated with an increased CgA fragmentation, particularly in the C-terminal region, as observed in patients.

### The Growth of Subcutaneous Panc02 Tumors Is Inhibited By Administration of Aprotinin or Anti-PGPQLR Antibodies

**Figure 5:**
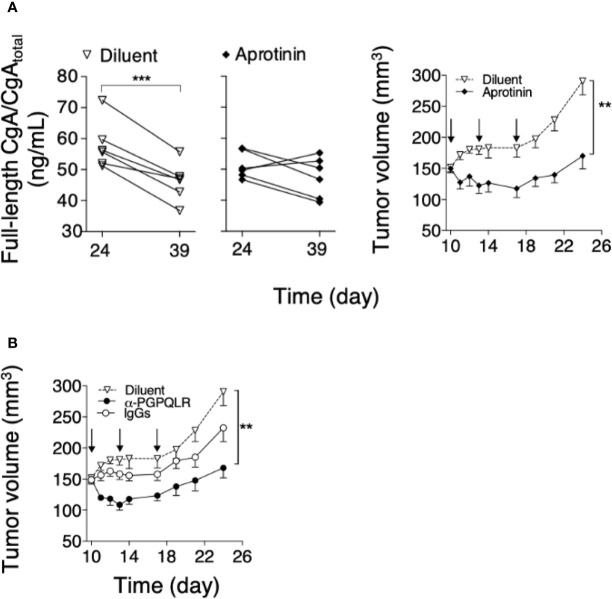
Effect of aprotinin or anti-PGPQLR antibodies on Panc0-tumor growth in mice. **(A)** Effect of aprotinin on the degradation of circulating CgA (*left*) and on the growth of Panc02-tumors (*right*). Panc02 tumor-bearing mice (n=6/group) were treated at day 10, 13 and 17 (*arrows*) with intra-peritoneal injections of aprotinin (100 μg/mouse) or diluent (saline solution). Plasma samples were collected from each mouse at day 24 and 39 and analyzed by full-length CgA–ELISA and CgA_total_–ELISA. The full-length CgA/CgA_total_ ratio (an index of C-terminal integrity) was then calculated (*left panels*); ***P <0.001 (paired *t*-test). Tumor volumes (*right panel*) were measured with calipers (mean ± SEM); **P <0.01 (unpaired *t*-test). **(B)** Effect of anti-PGPQLR (Pro-Gly-Pro-Gln-Leu-Arg) antibodies (capable of neutralizing the neuropilin-1 binding site of CgA_1-373_) on Panc02-tumor growth. Tumor-bearing mice were treated (i.p) at the indicated time (*arrows*) with 50 μg/mouse of rabbit anti-PGPQLR IgGs or control IgGs, or saline solution (Diluent). Tumor volumes (mean ± SEM); **P <0.01 (two tailed *t*-test).

To assess the hypothesis that CgA fragmentation has a role in the regulation of tumor progression, mice bearing subcutaneous Panc02 tumors were treated with aprotinin (to inhibit CgA fragmentation). A significant reduction of the full-length CgA/CgA_total_ ratio was observed from day 24 to 39 in mice treated with the diluent, pointing to CgA cleavage, but not in mice treated with aprotinin (**Figure 5A**, left). These data suggest a role of a serine protease, possibly plasmin, in CgA cleavage. Notably, aprotinin could also reduce the tumor growth rate (**Figure 5A**, right), suggesting that CgA fragmentation contributed to tumor growth regulation.

To assess this hypothesis, Panc02-bearing mice were injected with anti-PGPQLR IgGs, previously shown to a) neutralize the neuropilin-1 binding site of human CgA_1-373_ (PGPQLR_373_), b) cross-react with the corresponding site of its murine counterpart (100% conserved), and c) inhibit tumor growth in various murine models of solid tumors (8). Systemic administration of these antibodies, but not of normal rabbit IgGs, significantly reduced tumor growth (**Figure 5B**). As these antibodies cannot cross-react with the cryptic PGPQLR site of full-length CgA (8), these data support the hypothesis that CgA cleavage at this site contributed to tumor growth.

### Recombinant CgA Can Affect Tumor Perfusion in an Orthotopic Model

**Figure 6:**
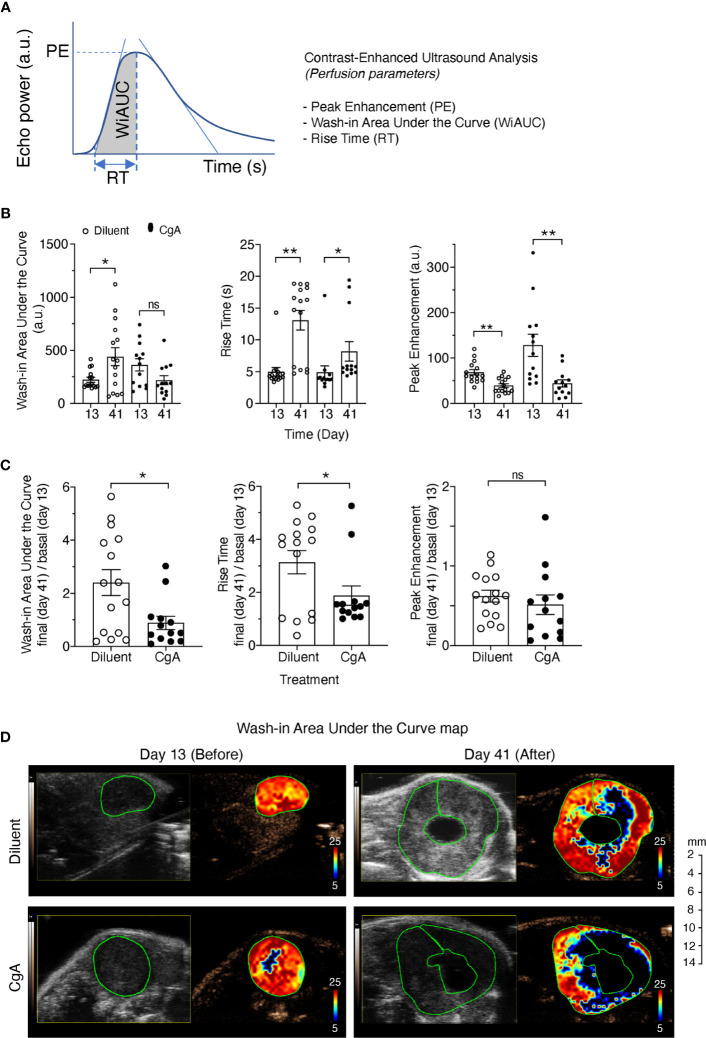
Effect of CgA on the perfusion of orthotopic DT6606 pancreatic ductal adenocarcinoma (PDAC). DT6606 cells were implanted in the pancreas of syngeneic mice and treated with recombinant CgA_1-439_ (1.5 µg/mouse, i.p. twice a week from day 14 to day 38, eight times) in 0.9% sodium chloride (Diluent) or with Diluent alone (n=15 mice/group). Tumor perfusion was monitored by contrast-enhanced ultrasound analysis one day before treatment (day 13) and three days after the last treatment (day 41). Perfusion parameters were calculated using a region of interest (ROI) corresponding to the entire tumor area [see **(A)** for a graph showing the parameters analyzed]. Perfusion parameters were: Peak Enhancement (PE, difference between the maximum amplitude in the curve and the baseline level, indicative of relative blood volume); Wash-in Area Under the Curve (WiAUC, area under the curve from starting enhancement to peak enhancement); rise time (RT, time frame during which intensity varies from 5%–95% of PE). **(B)** Tumor perfusion parameters at day 13 and 41 of mice treated with CgA or with Diluent. **(C)** Relative values of perfusion parameters at day 41 (final) over day 13 (basal). Outlier points were identified using the Prism software (ROUT, Q=5%) and excluded. *P <0.05; **P <0.01 (two-tailed *t*-test); ns: not significant. **(D)** Gray scale tumor images and color-coded Wash-in Area Under the Curve (WiAUC) maps of representative tumors (with intra-tumoral cystic lesions) at day 13 and day 41, treated with Diluent or CgA as indicated. Green lines indicate the ROI analyzed.

Finally, we investigated the effect of exogenous CgA on tumor vasculature and perfusion using DT6606 PDAC cells implanted orthotopically in the pancreas of syngeneic mice. To this aim, tumor-bearing mice were treated twice a week with 1.5 µg of full-length CgA (i.p.). Tumor perfusion was monitored one day before treatment (day 13) and three days after the last treatment (day 41), by contrast-enhanced ultrasound analysis with microbubbles. A significant decrease of the final/basal ratio of the wash-in area under the curve (WiAUC) and rise time (RT) of microbubbles was noted in animals treated with CgA (**Figure 6**). These data suggest that full-length CgA could affect the tumor vascular physiology and reduce perfusion of tumor tissues in this PDAC model.

## Discussion

The results show that fragmentation of the circulating pool of CgA is associated with shorter progression-free and overall survival in patients with PDAC. In particular, a decrease of full-length CgA and a relative increase of fragments lacking all or part of the C-terminal region (CgA_1-x_) were found in plasma samples of patients with worse prognosis (**Table 2**). Although the N-terminal fragment CgA_1-76_ was also detected in these patients, only the CgA_1-x_/CgA_total_ ratio, and not the CgA_1-76_/CgA_total_ (i.e., C- and N-terminal fragmentation indexes, respectively), was associated with patient prognosis. It appears, therefore, that fragmentation of the CgA C-terminal region is more relevant than fragmentation of the N-terminal region for patient prognosis.

Considering that administration of proton pump inhibitors is known to induce enterochromaffin-like cell hyperplasia and CgA secretion from the gastric mucosa (18, 19), the question arises as to whether these drugs induced the change in the circulating pool of full-length CgA and fragments observed in PDAC patients. This hypothesis is ruled out by the observation that patients taking proton pump inhibitors had a lower C-terminal fragmentation index, compared to untreated patients (**Table S3**). These and the above findings raise the question as to whether enhanced CgA cleavage in PDAC patients, e.g., at residue R_373_, occurred in neuroendocrine secretory cells (by intragranular or extracellular proteases), in the blood, or in tumors, by locally produced proteases. The results of a series of *in vitro* and *in vivo* studies performed with PDAC experimental models suggest that CgA fragments were produced in tumor tissues, by tumor-derived proteases. In particular, *in vitro* studies performed with various cultured human PDAC cell lines showed that these cells can promote the cleavage of exogenous full-length CgA added to the cell cultures (**Figure 1**). Interestingly, no cleavage was observed when plasminogen was omitted or when plasminogen activator inhibitor-1 was added to the cell cultures, pointing to an important role of plasminogen activators and plasmin in CgA cleavage. Accordingly, also aprotinin, an inhibitor of plasmin and other serine proteases, could inhibit CgA cleavage by these cells. CgA cleavage was observed also with cultured human monocyte-derived macrophages or with cancer-associated fibroblasts derived from PDAC. Although macrophages and cancer-associated fibroblasts required much longer time than cancer cells to induce CgA cleavage, also these cells might significantly contribute to CgA cleavage in patients, given their considerable abundance in the tumor stroma. Thus, various kinds of cells potentially present in the tumor tissue may contribute, to a different extent, to CgA cleavage.

This view is further supported by the results of *in vivo* studies, showing that the growth of murine Panc02, human BxPC3, or human Hs766T PDAC cells implanted subcutaneously in mice is associated with increased fragmentation of circulating CgA (**Figures 3** and **4**). Notably, a significant correlation between tumor volume and CgA cleavage was observed in the Panc02 and BxPC3 models, possibly because large tumors could release more proteolytic enzymes. Furthermore, in the Panc02 model, aprotinin could significantly inhibit CgA fragmentation (**Figure 5**), pointing again to a role of a serine protease in CgA cleavage.

The capability of plasmin to preferentially cleave the C-terminal region of CgA is supported by the results of SDS-PAGE, western blotting, and mass spectrometry analysis of CgA fragments obtained after limited or extensive digestion of recombinant CgA with plasmin (**Figure 2**). Indeed, analysis of the time course of CgA degradation with these techniques showed that certain sites located in the C-terminal region, e.g., at residue 355, 359, 373, and/or 394, are more prone to plasmin cleavage than sites located in the N-terminal region, the latter sites requiring a markedly longer time of incubation for cleavage. Thus, the results of *in vitro* and *in vivo* studies suggest that PDAC can promote the cleavage of CgA C-terminal region by activating plasminogen to plasmin, which in turn preferentially cleaves CgA in the C-terminal region. Interestingly, previous studies have shown that PDAC cells are characterized by increased expression of urokinase-type plasminogen activator (uPA) and uPA receptor, compared to normal pancreatic tissue, and by reduced expression of plasminogen activator inhibitor-1 (PAI-1) (20–22), indicating that indeed this proteolytic machinery is activated in PDAC. Of course, we cannot exclude that other proteases are also involved.

Given these premises, the question arises as to whether CgA cleavage is just an epiphenomenon of tumor growth or whether it can affect tumor physiology. The following observations suggest that CgA cleavage may contribute to regulate tumor growth. First, we have previously shown that CgA_1-439_ and CgA_1-76_ can regulate the adhesion of fibroblasts and endothelial cells, enhance the endothelial barrier function, and inhibit angiogenesis, whereas the fragment CgA_1-373_ can stimulate angiogenesis (5, 23, 24). In particular, full-length CgA and the N-terminal fragment CgA_1-76_ can inhibit endothelial cell migration, motility, sprouting, invasion and capillary-like structure formation induced by vascular endothelial growth factor, as well as angiogenesis induced by fibroblast growth factor-2 (7, 11). Vasostatin-1 can also inhibit, in endothelial cells, the nuclear translocation of hypoxia inducible factor-1α, a master regulator of angiogenesis (7, 11). On the other hand, other fragments, such as CgA_1-373_ and CgA_352-372_, can induce the release of fibroblast growth factor-2 from endothelial cells and promote angiogenesis (5, 14). Thus, CgA-related circulating polypeptides may form a balance of vasoactive factors, sometimes endowed of opposite effects, tightly regulated by N- and C-terminal proteolysis. According to this view, our results show that limited digestion of CgA with plasmin can progressively convert its anti-angiogenic into pro-angiogenic activity in the endothelial cell-spheroid capillary-sprouting assay, whereas extensive digestion abrogated both effects (**Figure 2C**). It appears, therefore, that CgA degradation by plasmin represents a mechanism for the fine regulation of PDAC vascular biology and growth. This hypothesis is further supported by the results of *in vivo* studies showing that inhibition of CgA cleavage in Panc02-bearing mice, by systemic administration of aprotinin, is associated with a lower tumor-growth rate. Furthermore, systemic administration of antibodies against the binding site of CgA_1-373_ for neuropilin-1 (PGPQLR), an important receptor for its pro-angiogenic activity (8), significantly reduced Panc02-tumor growth (**Figure 5**). Notably, these antibodies cannot react with the cryptic PGPQLR site of full-length CgA (8), suggesting that this epitope, exposed in fragments, might represent a therapeutic target for specific antibodies. Finally, the results of contrast-enhanced ultrasound analysis of tumors implanted in the pancreas of mice, showing that systemic administration of exogenous low-dose CgA_1-439_ can cause a significant reduction of tumor perfusion with microbubbles (**Figure 6**), lends further support to the hypothesis that this protein can indeed regulate the tumor vascular biology.

In conclusion, our findings suggest that PDAC can promote the cleavage of circulating CgA C-terminal region into vasoactive fragments, with important implications for PDAC pathophysiology and patient prognosis. Because of its potential pathophysiological function in the regulation of tumor biology and growth, CgA may represent a potential therapeutic target that warrants further exploration.

## Data Availability Statement

The raw data supporting the conclusions of this article will be made available by the authors, without undue reservation.

## Ethics Statement

The studies involving human participants were reviewed and approved by San Raffaele Hospital Ethics Committee. The patients/participants provided their written informed consent to participate in this study. The animal study was reviewed and approved by San Raffaele Hospital Ethics Committee and by Ministero della Salute of Italy.

## Author Contributions

Conception/design: MR, AC. Data acquisition: VA, AG, ED, BC, MM, MB, AD, ACi, EA, MP, and FC. Data analysis/interpretation: MR, VA, AMG, ED, MM, MB, AD, ACi, EA, MPP, AE, MF, FC, LP, and AC. Drafting: MR, VA, and AC. Critical revision: MR, VA, AG, ED, BC, MM, MB, AD, ACi, EA, MP, AE, MF, FC, LP, and AC. All authors contributed to the article and approved the submitted version.

## Funding

This work was supported by Associazione Italiana per la Ricerca sul Cancro (AIRC, grant IG-19220 and IG-23470, P.I. A Corti) and Fondazione AIRC under 5 per Mille 2019 – ID. 22737 program – P.I. MC Bonini, Group leader, A Corti.

## Conflict of Interest

The authors declare that the research was conducted in the absence of any commercial or financial relationships that could be construed as a potential conflict of interest.

## Abbreviations

CgA, chromogranin A; CTS, catestatin; PDAC, pancreatic ductal adenocarcinoma; PPI, proton pump inhibitors; PST, pancreastatin; SERP, serpinin; VS1, vasostatin-1.
