# Supplementary material for: Circulating Chromogranin A Is Cleaved Into Vasoregulatory Fragments in Patients With Pancreatic Ductal Adenocarcinoma
Source: Front Oncol. 2020 Dec 23;10:613582. doi: 10.3389/fonc.2020.613582 (PMC7787052; doi:10.3389/fonc.2020.613582)
Supplement: Supplementary file 1 [file DataSheet_1.docx]

Supplementary Material

# Supplementary Methods

*Antibodies and immunoassays*

Rabbit antibodies capable of recognizing the PGPQLR sequence of CgA_1-373_, but not that of CgA_1-439_, were produced as described previously and purified by affinity chromatography on Protein-A-Sepharose (8). Monoclonal antibodies (mAb) B4E11 and 5A8 against the epitopes 68-71 and 54-57, respectively, of full-length CgA and fragments were described previously(25, 26). Polyclonal antisera against human CgA_71-76_, CgA_368-373_, or CgA_410-439_, CgA_434-439_ (called α-76, α-373, α-410-439 and α-439, respectively) were raised in rabbits by immunization with the corresponding synthetic peptides coupled to keyhole limpet hemocyanin as described(8, 12). The antiserum α-76 binds CgA_1-76_, but not larger fragments; α-373 binds CgA_1-373_ and CgA_1-372_, but not larger precursors; α410-439 binds CgA_1-439_ and CgA_1-436_; α-439 binds CgA_1-439_, but not CgA_1-436_(8, 12). A polyclonal antiserum was also raised in rabbits by immunization with recombinant CgA_1-439_. This antiserum (called α-FRs) recognizes epitopes primarily located in the central region (immunodominant epitopes 90-133, 163-187, 222-256, 315-338) and recognizes, therefore, full-length CgA_1-439_ as well as various fragments containing the N-terminal region and part or the entire central region (e.g. CgA_1-436,_ CgA_1-409_, CgA_1-400_, CgA_1-373,_ CgA_1-372_ and shorter fragments), but not CgA_1-76_(8, 12).

Full-length CgA and CgA fragments were detected in plasma samples using five sandwich ELISAs based on the antibodies described above (see **Fig. S1** for a schematic representation of antibody epitopes and assays, and **Table S1** for analyte specificity of each assay)(8, 12). These assays can selectively detect: a) full-length CgA (CgA_1-439_−ELISA); b) full-length CgA with or without the C-terminal sequence 437-439 (CgA_1-436/439_−ELISA); c) CgA_1-372_ and CgA_1-373_ (CgA_1-372/373_−ELISA); d) CgA_1-76_ (CgA_1-76_−ELISA); e) full-length CgA and fragments containing the N-terminal region plus part or the entire central and C-terminal regions, but not CgA_1-76_ (defined here as “CgA_total_”) (CgA_total_−ELISA)(12).

The cumulative amounts of large fragments lacking the region 410-430 region (e.g. CgA_1-409_, CgA_1-394_, CgA_1-373_, plus other potential fragments with unknown C-terminus), were calculated as the difference between CgA_total_ and CgA_1-436/439_ and collectively called CgA_1-x_.

*Cells*

Human PDAC cells BxPC-3 (ATCC CRL-1687), Hs766T (ATCC HTB-134), MiaPaCa-2 (ATCC CRL-1420), PT45 (ATCC CRL-2558) and A8184 were cultured in RPMI 1640 supplemented with 10% fetal bovine serum, 2 mM glutamine, 100 µg/ml streptomycin, 100 U/ml penicillin and 0.25 µg/ml amphotericin-B. Murine PDAC cells DT6606 (obtained from a tumor arisen in a PdxCre/LSL-KrasG12D mouse)(27) were maintained in RPMI 1640 supplemented with 10% fetal bovine serum, 2 mM glutamine, 100 µg/ml streptomycin, 100 U/ml penicillin. Cancer associated fibroblasts were isolated from human PDAC pieces, obtained from two patients, put in culture in IMDM medium (Lonza) plus 10% FBS: cancer associated fibroblasts were obtained by outgrowth. Human monocytes were isolated from human peripheral blood mononuclear cells (PBMCs), by cell adhesion, and induced to differentiate to macrophages for 7 days with human macrophage colony stimulating factor. A vial of working cell bank was used to start new experiments; the cells were cultured for no more than 4 weeks before use. All cell lines were Mycoplasma-free, as routinely tested using the MycoAlert Control Set (Lonza).

*Endothelial spheroid capillary sprouting assay*

Human umbilical vein endothelial cells were purchased from Lonza and cultured as recommended by the manufacturer. Only endothelial cells cultured for less than eight passages were used. Endothelial cell spheroid assays were performed as described previously(6, 8). The number of pseudo-capillaries sprouting from each spheroid after 20 h of incubation with or without CgA or CgA fragments were counted using a microscope; to cumulate different experiments, data were normalized and expressed as % of control, i.e. the mean number of capillaries sprouting from spheroids treated with only the diluent of CgA.

# Supplementary Figures and Tables

## Supplementary Figures


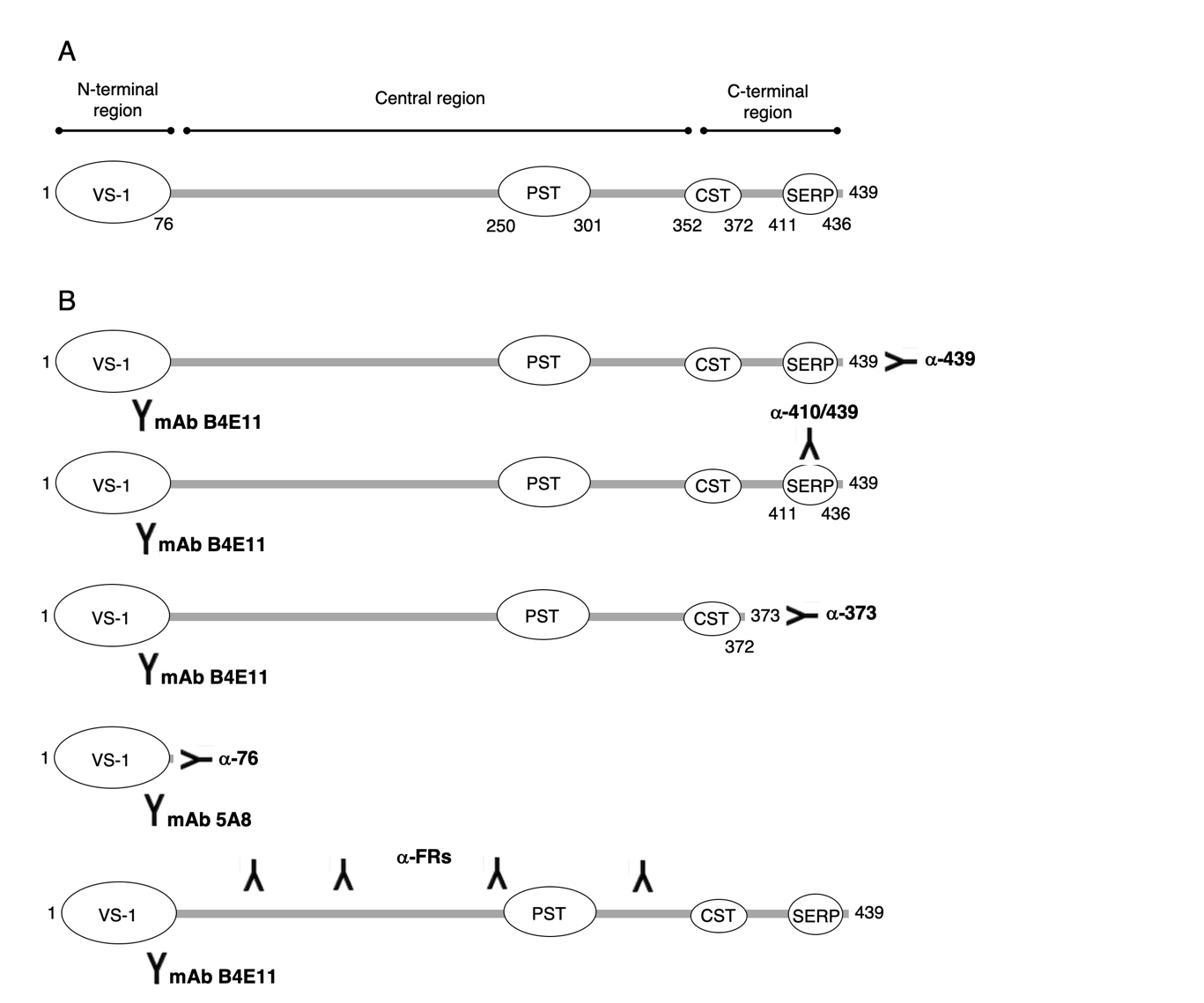


**Fig. S1.** **Schematic representation of CgA and ELISAs**

(A) Schematic representation of full-length human CgA and of its N-terminal, central and C-terminal regions. The regions corresponding to vasostatin-1 (VS-1), pancreastatin (PST), catestatin (CST) and serpinin (SERP) are indicated.

(B) Schematic representation of the five sandwich-ELISAs used in the present work (from top to bottom: CgA_1-439_−, CgA_1-436/439_−, CgA_1-372/373_−, CgA_1-76_− and CgA_total_−ELISA), based on antibodies against different CgA epitopes. In bold are indicated the antibodies used in the capture step (mAb B4E11 or 5A8) and detection step (α-439, α-410/439, α-372/373, α-76, and α-FRs) of the various assays.


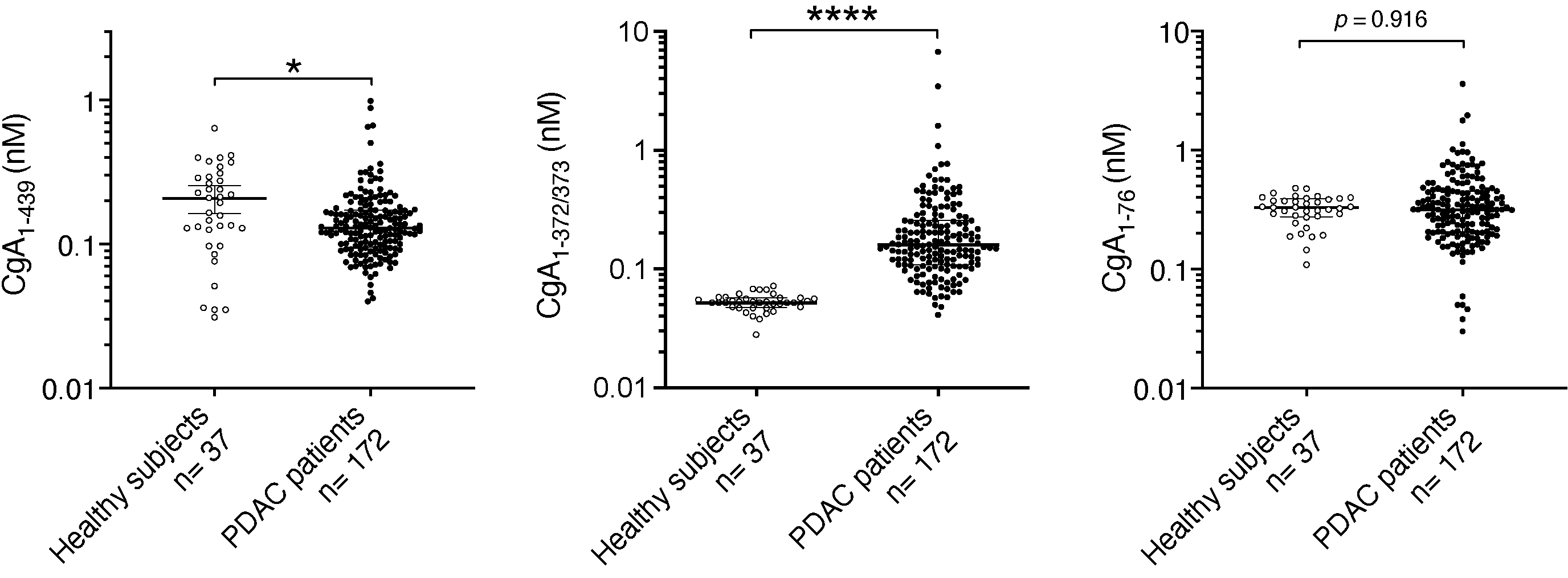


**Fig. S2. Plasma levels of CgA_1-439_, CgA_1-373_ and CgA_1-76_ in healthy subjects and PDAC patients**

**CgA_1-439_, CgA_1-373_ and CgA_1-76_** levels in healthy subjects (n=37) and in PDAC patients (n=172). ****, *P* <0.0001 (Mann-Whitney U test). Limit of detection: CgA_1-439_ (0.005 nM); CgA_1-373_ (0.040 nM); CgA_1-76_ (0.027 nM). Limit of quantification: CgA_1-439_ (0.017 nM); CgA_1-373_ (0.149 nM); CgA_1-76_ (0.063 nM) (28).

## Supplementary Tables

**Table S1. Analyte specificity of CgA-ELISAs based on different anti-CgA antibodies^a^**

| **ELISA** | **CgA forms detected** | | | | | |
| --- | --- | --- | --- | --- | --- | --- |
|  | ***CgA_1-439_*** | ***CgA_1-436_*** | ***CgA_1-372_*** | ***CgA_1-373_*** | ***CgA_1-x_^b^*** | ***CgA_1-76_*** |
| **CgA_1-439_−ELISA** | yes | no | no | no | no | no |
| **CgA_1-436/439_−ELISA** | yes | yes | no | no | no | no |
| **CgA_1-372/373_−ELISA** | no | no | yes | yes | NA^c^ | no |
| **CgA_1-76_−ELISA** | no | no | no | no | no | yes |
| **CgA_total_−ELISA** | yes | yes | yes | yes | yes | no |

a) See **Supplementary Methods** for the antibodies used in the different ELISAs and their epitopes, and **Fig. S1** for a schematic representation of each assay.

b) x: any residue of the 133-410 region. Thus, CgA_1-x_ includes fragments of different length containing the N-terminal region 1-76 plus part of the central/C-terminal region and lacking the 410-439 region. CgA_1-x_ is calculated as the difference between the antigen detected by CgA_total_−ELISA and CgA_1-436/439_−ELISA (see **Fig. S1**)

c) NA, not applicable: this assay can detect fragments CgA_1-372/373_, which belong to the CgA_1-x_ family, but not larger or shorter fragments.

| **Table S2.** **Demographic and clinical details regarding the study population.** | | |
| --- | --- | --- |
| **Variable** | ***PDAC patients***  ***(n=172)*** | ***Heathy subjects***  ***(n=37)*** |
| **Age**  Median  25^th^- 75^th^  Min-max | **years**  60  54-66  32-75 | **years**  61  51-71  28-79 |
| **Gender**  Female  Male | **n (*%*)**  85 (*49)*  87 (*51*) | **n (*%*)**  13 (*35*)  24(*65*) |
| **Karnofsky Performance Status (%)**  70  80  90  100 | 10 (*6*)  28 (*16)*  88 (*51*)  46 (*27*) | -  -  -  - |
| **Stage**  III  IV | 65 (*38*)  107 (*62*) | -  - |
| **Proton pump inhibitor therapy**  No  Yes | 75 (*44*)  97 (*56*) | -  - |
| **Chemotherapy regimen**^a^  PDXG  PEXG | 24 (*14*)  148 (*86*) | -  - |
| ^a)^ cisplatin (P); docetaxel (D), capecitabine (X), gemcitabine (G), epirubicin (E) | |  |

| **Table S3. Plasma levels of CgA and fragments in patients assuming and not assuming proton pump inhibitors.** | | | |  |
| --- | --- | --- | --- | --- |
|  | **Non-PPI users**^a^  *n = 75* | **PPI users**  *n = 97* | ***P value*** | |
| **CgA_total_ (nM)**  Median  25^th^-75^th^  Min-max | 0.86  0.54-1.64  0.22-14.28 | 0.97  0.67-1.57  0.31-10.00 | *0.184* | |
| **CgA_1-439_ (nM)**  Median  25^th^-75^th^  Min-max | 0.13  0.10-0.16  0.04-0.99 | 0.13  0.09-0.18  0.05-0.67 | *0.938* | |
| **CgA_1-436/439_ (nM)**  Median  25^th^-75^th^  Min-max | 0.42  0.25-0.76  0.05-2.35 | 0.49  0.37-0.82  0.11-3.71 | ***0.042*** | |
| **CgA_1-x_ (nM)**  Median  25^th^-75^th^  Min-max | 0.41  0.26-0.89  0.02-11.93 | 0.45  0.25-0.85  0.001-7.32 | *0.900* | |
| **CgA_1-372/373_ (nM)**  Median  25^th^-75^th^  Min-max | 0.17  0.12-0.24  0.04-6.73 | 0.15  0.11-0.28  0.05-3.46 | *0.480* | |
| **CgA_1-76_ (nM)**  Median  25^th^-75^th^  Min-max | 0.32  0.21-0.44  0.05-1.02 | 0.32  0.21-0.51  0.01-3.61 | *0.634* | |
| **CgA_1-x_/CgA_total_**  Median  25^th^-75^th^  Min-max | 0.54  0.45-0.62  0.05-0.87 | 0.49  0.38-0.61  0.001-0.97 | ***0.032*** | |
| **CgA_1-439_ / CgA_total_**  Median  25^th^-75^th^  Min-max | 0.13  0.09-0.23  0.04-0.45 | 0.12  0.09-0.19  0.01-0.49 | *0.304* | |
| ^a^PPI, proton pump inhibitors | | | |  |

| **Table S4.** **Follow up data, progression free and overall survival of patients with locally advanced or metastatic pancreatic ductal adenocarcinoma (n=172).** | |
| --- | --- |
| **Variable** |  |
| **Progression**  No  Yes | **n (%)**  4 (2)  168 (98) |
| **Death**  No  Yes | 4 (2)  168 (98) |
|  | **Months** |
| **Progression-free survival**  Median  25^th^-75^th^ | 8  4-11 |
| **Overall survival**  Median  25^th^-75^th^ | 12  8-19 |
|  | |

| **Table S5. Univariate Cox-regression analysis evaluating predictors of progression-free and overall survival in patients with locally advanced or metastatic pancreatic ductal adenocarcinoma (n=172).** | | | | | | | |
| --- | --- | --- | --- | --- | --- | --- | --- |
|  | **Progression-free survival** | | |  | **Overall survival** | | |
| **Variable** | **HR**^a^ | **95% C.I.** | ***P*** |  | **HR**^a^ | **95% C.I.** | ***P*** |
| **Sex**  Female  Male | 1  1.084 | -  0.800-1.469 | *0.604* |  | 1  1.262 | -  0.931-1.711 | *0.133* |
| **Age**  ≤ 60 years  > 60 years | 1  0.745 | -  0.549-1.010 | *0.058* |  | 1  0.820 | -  0.605-1.112 | *0.201* |
| **Stage**  III  IV | 1  2.359 | -  1.707-3.261 | ***<0.0001*** |  | 1  1.898 | -  1.384-2.604 | ***<0.0001*** |
| **Karnofsky performance status** | 0.969 | 0.951-0.988 | ***0.001*** |  | 0.972 | 0.954-0.990 | ***0.003*** |
| **CA 19.9** | 1.020 | 1.009.1.031 | ***<0.0001*** |  | 1.025 | 1.014-1.037 | ***<0.0001*** |
| **Chemotherapy regimen**  PDXG  PEXG | 1  1.354 | -  0.859-2.133 | *0.192* |  | 1  1.085 | -  0.692-1.701 | *0.723* |
| **Proton pump inhibitors**  No  Yes | 1  0.932 | -  0.686-1.266 | *0.652* |  | 1  1.005 | -  0.740-1.366 | *0.972* |
| **CgA_total_** | 1.038 | 0.948-1.136 | *0.418* |  | 1.009 | 0.923-1.102 | *0.851* |
| **CgA_1-439_** | 0.942 | 0.264-3.358 | *0.927* |  | 0.461 | 0.121-1.751 | *0.255* |
| **CgA_1-436/439_** | 1.121 | 0.861-1.459 | *0.397* |  | 1.009 | 0.780-1.306 | *0.944* |
| **CgA_1-x_** | 1.040 | 0.926-1.168 | *0.508* |  | 1.013 | 0.902-1.137 | *0.830* |
| **CgA_1-372/373_** | 1.130 | 0.898-1.422 | *0.297* |  | 1.051 | 0.817-1.352 | *0.699* |
| **CgA_1-76_** | 0.815 | 0.526-1.264 | *0.361* |  | 0.914 | 0.582-1.436 | *0.697* |
| **CgA_1-x_/CgA_total_** | 1.543 | 0.633-3.760 | *0.340* |  | 1.732 | 0.697-4.301 | *0.237* |
| **CgA_1-439_ / CgA_total_** | 0.542 | 0.098-2.979 | *0.481* |  | 0.285 | 0.049-1.643 | *0.160* |
| Abbreviations: PDXG, cisplatin+docetaxel+capecitabine+gemcitabine; PEXG, cisplatin+epirubicin+capecitabine+gemcitabine; HR, hazard ratio; C.I., confidence interval  ^a)^ Hazard ratio (HR) for every increase of: 10% (performance status); 1000 U/mL (CA 19.9); 1 nM (CgA_total_, CgA_1-439_, CgA_1-436/439_, CgA_1-x_, CgA_1-372/373_, CgA_1-76_); 1 unit (CgA_1-x_/CgA_total_, CgA_1-439_/CgA_total_). | | | | | | | |
